# Artificial Intelligence in FIT‐Positive Colonoscopy: Balancing Detection Metrics and Clinical Impact

**DOI:** 10.1002/ueg2.70193

**Published:** 2026-02-27

**Authors:** Ignasi Puig, Maria Pellisé

**Affiliations:** ^1^ Institut de Recerca i Innovació en Ciències de la Vida ide la Salut a la Catalunya Central (IRIS‐CC) Vic Spain; ^2^ Digestive Diseases Department Althaia Xarxa Assistencial Universitària de Manresa Manresa Spain; ^3^ Facultat de Medicina Universitat de Vic‐Central de Cataluña (UVIC‐UCC) Vic Spain; ^4^ Department of Gastroenterology Hospital Clínic Barcelona Spain; ^5^ Fundació de Recerca Clínic Barcelona‐Institut d'Investigacions Biomèdiques August Pi i Sunyer (FRCB‐IDIBAPS) Centro de Investigación Biomédica en Red de Enfermedades Hepáticas y Digestivas (CIBEREHD) Barcelona Spain; ^6^ Facultat de Medicina i Ciències de la Salud Universitatde Barcelona (UB) Barcelona Spain

Organised colorectal cancer (CRC) screening programs based on faecal immunochemical testing (FIT) have consistently demonstrated a reduction in CRC incidence and mortality. Colonoscopy following a positive FIT represents a critical step in this pathway, as it targets a population with a higher prevalence of advanced neoplasia and one in which limitations in colonoscopy performance translate into a higher risk of post‐colonoscopy colorectal cancer (PCCRC) [1]. In this demanding clinical scenario, improving colonoscopy performance remains a priority, and artificial intelligence–based computer‐aided detection (CADe) systems have emerged as a promising adjunct to enhance lesion detection [2].

Whilst CADe has been shown to improve adenoma detection rate (ADR) in average‐risk screening populations, its role in FIT‐positive colonoscopy remains uncertain. Meta‐analyses suggest that the incremental benefit of CADe may be attenuated in FIT‐positive cohorts with high baseline detection rates [3]. In this context, the multicentre randomised controlled trial by Spada and colleagues provides timely real‐world evidence from a FIT‐based screening programme [2].

In more than 1000 FIT‐positive individuals randomised to CADe‐assisted or standard colonoscopy, no difference was observed in the primary endpoint of advanced adenoma detection rate (AADR) [2]. By contrast, CADe significantly increased ADR and adenomas per colonoscopy (APC), with a statistically significant but clinically negligible increase in withdrawal time, no increase in the resection of non‐neoplastic lesions, and no safety signal [2]. These findings highlight both the potential and the limitations of CADe in this setting. As in many procedural trials, heightened operator awareness related to study participation may have contributed to the high baseline performance observed in both arms, limiting additional gains in clinically relevant detection.

Importantly, these findings are consistent with those of the CADILLAC trial, the largest randomised study conducted to date in FIT‐positive individuals [4]. In this adequately powered multicentre trial, CADe did not increase detection of advanced colorectal neoplasia, despite modest increases in small, proximal, and non‐polypoid lesions. Together, CADILLAC and the present study suggest that, in FIT‐based screening populations with high baseline performance, CADe may primarily increase detection of low‐risk lesions without improving detection of clinically relevant neoplasia [2, 4].

Advanced adenomas are most closely linked to future CRC risk, making AADR a particularly relevant outcome in FIT‐positive colonoscopy. Population‐based studies have demonstrated a strong inverse association between ADR and PCCRC risk in FIT‐based screening programs [1, 5]. The absence of an effect on AADR aligns with previous evidence suggesting that CADe has limited impact on advanced neoplasia detection when baseline performance is high [3]. In such settings, a “ceiling effect” may constrain further improvements in clinically meaningful outcomes.

By contrast, the observed increase in ADR and APC confirms that CADe consistently identifies additional lesions, particularly diminutive adenomas. This raises a key conceptual issue: are we measuring the outcomes that matter most? Although ADR remains a cornerstone quality indicator, it is intrinsically binary and susceptible to the “one‐and‐done” phenomenon. APC has therefore been proposed as a complementary metric to better capture inspection thoroughness in high‐ADR settings [6]. In the present trial, CADe significantly increased APC, suggesting a genuine effect on detection behaviour [2].

Nevertheless, the clinical implications of detecting more small, non‐advanced adenomas in FIT‐positive individuals remain uncertain. [7] These lesions carry limited short‐term malignant potential, and increased detection may translate into higher surveillance intensity and resource utilisation. Microsimulation models suggest that widespread CADe implementation could substantially increase surveillance colonoscopy burden, with an uncertain net effect on CRC outcomes [8].

This balance between modest potential benefits and unintended consequences is reflected in the 2025 European Society of Gastrointestinal Endoscopy (ESGE) Position Statement on CADe use during colonoscopy [9]. Based on randomised trials, microsimulation modelling, and patient values and preferences, the ESGE issued a weak recommendation in favour of CADe, acknowledging limited and uncertain impact on CRC incidence and mortality alongside increased detection and surveillance burden. Recent FIT‐based randomised trials such as those by Spada et al. [2] and CADILLAC [4] provide key empirical support for a cautious, value‐based implementation of artificial intelligence in organised screening programmes.

Beyond detection metrics, emerging data suggest that artificial intelligence may also influence endoscopist behaviour. An observational study reported a reduction in ADR during non–AI‐assisted colonoscopies following routine CADe exposure, raising concerns about potential deskilling effects [10]. Although these findings should be interpreted cautiously, they indicate that AI may reshape endoscopist behaviour beyond immediate detection gains.

Taken together, these data recalibrate expectations regarding the role of CADe in FIT‐positive colonoscopy. Artificial intelligence is not a universal solution, but a context‐sensitive tool whose value depends on baseline performance, operator expertise, and clinically meaningful outcomes. Ongoing large‐scale implementation studies, such as ACCEPT, will be essential to determine whether improvements in detection metrics translate into meaningful reductions in CRC incidence and mortality (ACCEPT study; UMIN000051108).

## Conflicts of Interest

The authors declare no conflicts of interest.

## Data Availability

Data sharing not applicable to this article as no datasets were generated or analysed during the current study.
